# Prevalence of resurgence of destructive behavior when thinning reinforcement schedules during functional communication training

**DOI:** 10.1002/jaba.472

**Published:** 2018-05-17

**Authors:** Adam M. Briggs, Wayne W. Fisher, Brian D. Greer, Ryan T. Kimball

**Affiliations:** ^1^ University of Nebraska Medical Center's Munroe‐Meyer Institute

**Keywords:** differential reinforcement, extinction burst, functional communication training, reinforcement schedule thinning, resurgence

## Abstract

Functional communication training is a well‐established treatment for socially reinforced destructive behavior that typically includes differential reinforcement of the functional communication response (FCR) in combination with extinction of destructive behavior. However, when the schedule of reinforcement for the FCR is thinned, destructive behavior may resurge (e.g., Greer, Fisher, Saini, Owen, & Jones, 2016). Currently, data are unavailable on the prevalence and characteristics of resurgence during reinforcement schedule thinning. In this study, we evaluated the prevalence of resurgence during reinforcement schedule thinning on a per‐case and per‐schedule‐step basis and also evaluated the magnitude of resurgence in relation to the functions of destructive behavior. We observed resurgence in 19 of the 25 (76%) applications of reinforcement schedule thinning. In some cases, the magnitude of resurgence exceeded the mean levels of destructive behavior observed in baseline. We discuss these results relative to prior translational and applied research on resurgence.

Functional communication training (FCT; Carr & Durand, 1985) involves the delivery of the reinforcer responsible for maintenance of destructive behavior contingent on an alternative communication response, usually in combination with extinction of destructive behavior. Prior research has found FCT to be a well‐established treatment for a variety of topographies of socially reinforced destructive behavior (e.g., aggression, self‐injurious behavior; Greer, Fisher, Saini, Owen, & Jones, 2016; Hagopian, Fisher, Thibault‐Sullivan, Acquisto, & LeBlanc, 1998; Kurtz, Boelter, Jarmolowicz, Chin, & Hagopian, 2011). Functional communication training generally proceeds according to the following treatment sequence. First, the behavior analyst conducts a functional analysis (FA) to identify the reinforcing consequence(s) for destructive behavior. Second, the analyst prompts and reinforces a functionally equivalent communication response (FCR), while exposing destructive behavior to extinction, until the individual emits the FCR independently in the presence of the relevant establishing operation. Finally, the analyst thins the schedule of reinforcement until the terminal schedule approximates the practical constraints of the individual's natural environment (e.g., so that the parent can reasonably implement the treatment in the home while simultaneously completing other family responsibilities). For more information regarding the progression described above, see Fisher and Bouxsein (2011), Fisher, Greer, and Fuhrman (2015), Hagopian, Boelter, and Jarmolowicz (2011), and Tiger, Hanley, and Bruzek (2008).

Prior research has demonstrated that discontinuation of reinforcement for destructive behavior, or extinction, during FCT is often important for the treatment's effectiveness (e.g., Hagopian et al., 1998; Shirley, Iwata, Kahng, Mazaleski, & Lerman, 1997; cf. Athens & Vollmer, 2010). In a recent summary of the effects of FCT with a series of 25 successive applications in a well‐defined cohort of patients, Greer, Fisher, Saini, et al. (2016) observed that FCT, when combined with extinction, reduced destructive behavior by an average of 92% relative to baseline levels. In addition, Greer, Fisher, Saini, et al. successfully reached the target schedule of FCR extinction periods lasting at least 4 min (or 8 min when they programmed extinction [S^Δ^] periods back‐to‐back) while maintaining low rates of destructive behavior in 88% of applications. Finally, the investigators found it necessary to add supplemental procedures to 28% of applications, producing a reduction in destructive behavior by an average of 96% relative to baseline levels.

Although treatments involving extinction, such as FCT, are often effective, they also may be associated with side effects, including extinction bursts (Lerman & Iwata, 1995), extinction‐induced aggression (Lerman, Iwata, & Wallace, 1999), and resurgence of destructive behavior (Fuhrman, Fisher, & Greer, 2016; Mace et al., 2010; Marsteller & St. Peter, 2012; Volkert, Lerman, Call, & Trosclair‐Lasserre, 2009; Wacker et al., 2011; Wacker et al., 2013). Specifically, prevalence studies found that when extinction‐based interventions were implemented in isolation, bursts of self‐injurious behavior and extinction‐induced aggression occurred in 43% and 29% of applications, respectively; however, when combining extinction with other procedures, such as differential reinforcement, the number of applications with either of these side effects reduced by more than 50% (Lerman & Iwata, 1995; Lerman et al., 1999). In addition, Fisher and colleagues showed that short exposures to the establishing operation for destructive behavior prevented extinction bursts, and longer exposures promoted extinction bursts (DeRosa, Fisher, & Steege, 2015; Fisher et al., 2018). These data help to clarify and describe two side effects of extinction, bursting and induced aggression, and identify variables that promote and diminish these side effects. However, little is known about the prevalence and characteristics of a third major side effect of extinction, resurgence of destructive behavior.

Resurgence is defined as the reemergence of a previously extinguished response (e.g., destructive behavior) when the alternative response is exposed to extinction or large decreases in the rate of reinforcement (Doughty & Oken, 2008; Epstein, 1983, 1985; Lattal et al., 2017; Lattal & St. Peter Pipkin, 2009; Leitenberg, Rawson, & Bath, 1970; Lieving, Hagopian, Long, & O'Connor, 2004; Lieving & Lattal, 2003; Podlesnik & DeLeon, 2015; Pritchard, Hoerger, & Mace, 2014; Winterbauer & Bouton, 2010). The study of resurgence represents an important area of investigation in both applied and translational research because it may form the basis of many or most occurrences of treatment relapse in typical environments (Kestner & Peterson, 2017; Lattal & St. Peter Pipkin, 2009; St. Peter, 2015). For example, individuals often emit the FCR at high rates or at inopportune times, when it is difficult for caregivers to deliver the requested reinforcer (Fisher et al., 1993; Hagopian et al., 1998; Tiger, Hanley, & Heal, 2006). During these situations, caregivers may expose the FCR to extinction or exceedingly lean schedules of reinforcement, which may result in resurgence of destructive behavior (e.g., Marsteller & St. Peter, 2012; Volkert et al., 2009).

In the typical treatment sequence described above for FCT, the FCR is likely to first meet a challenge that may result in resurgence of destructive behavior during reinforcement schedule thinning (Saini, Miller, & Fisher, 2016). That is, during reinforcement schedule thinning, the behavior analyst introduces periods in which reinforcement for the FCR is unavailable. For example, Greer, Fisher, Saini, et al. (2016) introduced periods in which the FCR was placed on extinction using either multiple schedules (when treating destructive behavior maintained by social‐positive reinforcement) or chained schedules (when treating destructive behavior maintained by social‐negative reinforcement). Visual inspection of Greer, Fisher, Saini, et al.’s four case examples showed resurgence of destructive behavior at least once during reinforcement schedule thinning. However, Greer, Fisher, Saini, et al. did not discuss these instances of resurgence, nor did they present data on how often resurgence occurred in their analysis of the effectiveness of FCT during reinforcement schedule thinning. Therefore, the purpose of the current investigation was to conduct a detailed examination of all of the data sets for all of the participants from the Greer, Fisher, Saini, et al. investigation to determine the prevalence and characteristics of resurgence during reinforcement schedule thinning in a relatively large and well‐defined cohort of participants.

## METHOD

### *Participants and Setting*

Participants in Greer, Fisher, Saini, et al. (2016) consisted of 20 individuals who averaged 7.5 years of age (range, 2‐19 years old). Most carried a diagnosis of an intellectual disability, and all were referred for the treatment of severe destructive behavior (see Table 1 in Greer, Fisher, Saini, et al., 2016, for participant ages, diagnoses, level of intellectual disability, and destructive behaviors). Twenty‐five consecutive applications of FCT schedule thinning, totaling 111 dense‐to‐lean transitions (defined under Response Measurement), were evaluated across the 20 participants. We defined an application of FCT schedule thinning as a case in which an FA indicated that destructive behavior was maintained by socially mediated consequences, and FCT was evaluated using signaled components to indicate when reinforcement was and was not available during reinforcement schedule thinning. If results of the FA indicated that destructive behavior was maintained by multiple reinforcers, separate applications of FCT and schedule thinning were sometimes conducted and were included as separate applications (see Tables 2 & 3 in Greer, Fisher, Saini, et al., 2016, for functions and schedule thinning procedures). All sessions took place in therapy rooms at a university‐affiliated program that specializes in the assessment and treatment of severe destructive behavior.

### *General Assessment and Treatment Procedures Implemented by Greer, Fisher, Saini, et al. (* 2016)

Greer, Fisher, Saini, et al. (2016) conducted pretreatment FAs for all participants, and results indicated reinforcement of destructive behavior by access to tangible consequences (9 of 25 applications), escape (8 of 25 applications), attention (4 of 25 applications), social control (3 of 25 applications), and attention plus tangible consequences (1 of 25 applications; see Table 2 in Greer, Fisher, Saini, et al.). Following the FA, Greer, Fisher, Saini, et al. evaluated the effects of FCT in comparison with the baseline condition. The condition (or conditions if destructive behavior served multiple functions) with the highest rates of destructive behavior during the FA served as the baseline condition during the FCT treatment evaluation. Following this baseline, Greer, Fisher, Saini, et al. trained the participant to emit the FCR independently in one or more pretreatment sessions using differential reinforcement with prompts and prompt‐fading procedures, which varied across participants. Following this pretraining, Greer, Fisher, Saini, et al. implemented FCT by delivering the functional reinforcer for the FCR on a fixed‐ratio (FR) 1 schedule and implemented extinction for destructive behavior. In most applications, Greer, Fisher, Saini, et al. evaluated the effects of FCT by alternating the baseline and FCT conditions in a reversal design prior to schedule thinning.

After evaluating FCT under optimal conditions, an FR‐1 schedule of reinforcement for FCRs, Greer, Fisher, Saini, et al. (2016) initiated reinforcement schedule thinning to make the treatment more practical for caregivers to implement under naturalistic conditions. They implemented schedule thinning during FCT using one of three procedures. FCT schedule thinning consisted of (a) multiple schedules (14 of 25 applications); (b) response restriction (RR) FCT, in which researchers removed the FCR response card during S^Δ^ periods (7 of 25 applications); or (c) chained schedules, which they switched to multiple schedules at the completion of schedule thinning (4 of 25 applications). In each of these three procedures, researchers alternated signaled periods in which the FCR produced reinforcement (S^D^ periods) with signaled periods in which the FCR (when available) produced no programmed consequence (S^Δ^ periods), and they either gradually (*n* = 10 of 25 applications) or rapidly (*n* = 15 of 25 applications) increased the duration of the extinction component. In addition, Greer, Fisher, Saini, et al. implemented differential reinforcement of other behavior (DRO) in one application of a multiple schedule and two applications of RR to prevent adventitious reinforcement.

### *General Record Review Procedures for Current Analyses*

We conducted several additional analyses from the case records originally compiled by Greer, Fisher, Saini, et al. (2016). Specifically, we extended this previous record review by analyzing the rate of destructive behavior for each application of FCT schedule thinning to determine whether and to what extent transitions from relatively dense to relatively lean schedules of reinforcement produced resurgence of destructive behavior. We identified transitions from dense‐to‐lean schedules of reinforcement by reviewing each step of reinforcement schedule thinning in relation to the prior step. We included only those transitions in which there was a decrement in the programmed rate (i.e., opportunity for reinforcer delivery), magnitude (i.e., duration of reinforcer access), or quality (i.e., presence of alternative reinforcers) from one schedule step to the next (i.e., we excluded transitions in which the programmed rate, magnitude, or quality of reinforcement increased and ones in which the programmed reinforcement remained the same while some other variable changed; e.g., introduction of a novel therapist).

### *Response Measurement*

We defined a *schedule‐thinning transition* as a change from one schedule step (Condition A) to the next schedule step (Condition B), in which the programmed rate, magnitude, or quality of reinforcement per session decreased in Condition B relative to Condition A. Specifically, schedule‐thinning transitions from Condition A to Condition B consisted of either decreasing the rate of reinforcement by (a) increasing the S^Δ^ duration (e.g., 30 s to 60 s in multiple‐schedule FCT); (b) increasing the response requirement (e.g., FR 1 to FR 2 in chained‐schedule FCT); (c) increasing the differential reinforcement interval (e.g., 5 s to 10 s in DRO); or (d) decreasing the S^D^ duration (e.g., 60 s to 30 s in multiple‐schedule FCT), or decreasing the magnitude (e.g., 60 s of iPad access to 30 s of iPad access) or quality (e.g., 30 s of escape with iPad access to 30 s of escape without iPad access) of reinforcement. For example, we considered a transition from a multiple‐schedule FCT 60/60 (seconds in S^D^/seconds in S^Δ^) in Condition A to a multiple‐schedule FCT 60/240 in Condition B to be a schedule‐thinning transition and therefore included it in our analysis.

After identifying the schedule‐thinning transitions that met our inclusion criteria, raters scored each transition for the presence or absence of resurgence based on the criteria described by Lerman and Iwata (1995) for identifying extinction bursts. Specifically, we defined *resurgence* as an increase in responding during any of the first three sessions of Condition B (or all of Condition B if it lasted fewer than three sessions) above that observed during any of the last five sessions of Condition A (or all of Condition A if it lasted fewer than five sessions). To determine this, raters extracted the rates of destructive behavior from the data summary for each session identified within a schedule‐thinning transition and organized the data into Condition A or Condition B. If any of the session rates of destructive behavior from Condition B exceeded any of the session rates of destructive behavior from Condition A, the rater scored this as a transition with resurgence. For example, if the first three sessions of Condition B produced rates of the destructive behavior of 2.4, 0.4, and 0.2 responses per minute, and the last five sessions of Condition A produced rates of 0, 0, 0.2, 0.4, and 0 responses per minute, we would score it as a transition with resurgence. In this case, Condition B produced a response rate (2.4) that exceeded the highest rate scored in Condition A (0.4). However, had Condition B produced rates of the destructive behavior of 0.4, 0.4, and 0.2 responses per minute, we would not score that as a transition with resurgence because Condition B did not produce a response rate that exceeded the highest rate scored in Condition A.

After reviewing all schedule‐thinning transitions and scoring them for the presence or absence of resurgence, we analyzed the magnitude of resurgence during transitions across applications. For each transition in which we identified resurgence, we identified the highest rate of destructive behavior from each condition (i.e., Conditions A and B of a transition) and converted each of these rates to a proportion of baseline to allow for a comparison of the magnitude of resurgence between conditions and across applications. We did this by dividing the highest rates of destructive behavior observed in Condition A and Condition B by the mean rate of destructive behavior scored over the last five sessions of baseline (or all sessions if baseline lasted fewer than five sessions). For this calculation, baseline consisted of the most recent phase in which destructive behavior produced reinforcement.

### *Interrater Agreement*

A second independent rater scored 8 of the 25 applications (32%) for the identification of (a) schedule‐thinning transitions, (b) resurgence during transitions, and (c) magnitude of resurgence during applicable transitions. First, each rater independently examined the graph and the data summary for each application of schedule thinning and scored (a) the frequency of schedule‐thinning transitions and (b) whether resurgence occurred during each schedule‐thinning transition based on the definition above. After independent evaluations of each application, we assessed item‐by‐item agreement on an occurrence/nonoccurrence basis by comparing data tables generated for each application by the two raters. We calculated interrater agreement by dividing the number of agreements by the number of agreements plus disagreements and converting the resulting proportion to a percentage for each measure within each application, resulting in a mean interrater agreement of 97% (range, 88%–100%) for frequency of transitions and 93% (range, 67%–100%) for resurgence during transitions across applications.

Second, we used the data summary from the applications in which we identified resurgence to calculate agreement on the magnitude of resurgence during transitions. Each rater independently calculated proportion of baseline (described above). Proportional agreement was assessed by dividing the smaller calculation by the larger calculation for each schedule‐thinning transition. We then averaged these proportional agreements across schedule‐thinning transitions for each application and multiplied by 100. Interrater agreement averaged 98% (range, 90%–100%) across applications. Finally, we reconciled all disagreements after calculating interrater agreement. Specifically, if interrater agreement was less than 100%, raters met to review the discrepancies and determine their sources. Discrepancies were infrequent but included (a) identifying an additional transition or instance of resurgence, (b) failing to identify a transition or instance of resurgence, or (c) incorrectly calculating the proportion of baseline for the magnitude of resurgence following their IOA calculations. Following a joint review of the disagreement, we reconciled the discrepancy and made necessary updates to the results as needed.

## RESULTS

**Figure 1:**
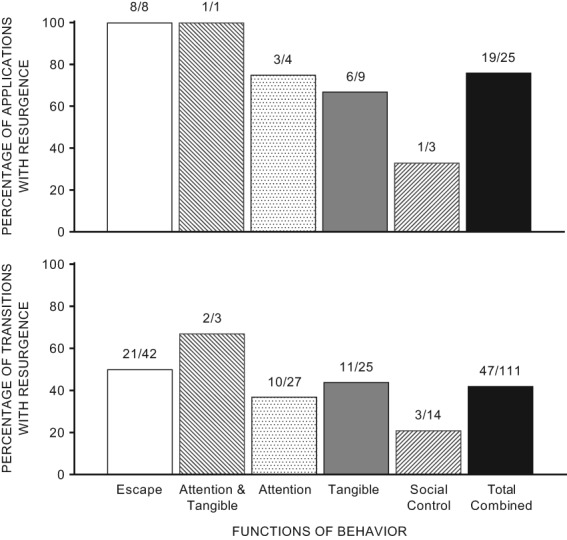
Prevalence of resurgence across applications of schedule thinning (top panel) and within dense‐to‐lean transitions throughout schedule thinning (bottom panel) organized by function of destructive behavior. Applications with resurgence (represented as numerator of fraction) and total applications for a given column (represented as denominator of fraction) highlight the varying number of applications within each function.

Figure 1 shows the number and percentage of the 25 applications of reinforcement schedule thinning in which we observed resurgence of destructive behavior during one or more transitions from a dense to a relatively leaner schedule of reinforcement for the FCR, organized by operant function (top panel). We observed resurgence in 19 of the 25 applications (76%) of reinforcement schedule thinning across all functions of destructive behavior. When examining correlates of resurgence, we found that destructive behavior resurged at least once in every application of reinforcement schedule thinning for destructive behavior reinforced by escape (8 of 8 applications; 100%) or attention plus tangible consequences (1 of 1 application; 100%), followed by attention (3 of 4 applications; 75%), tangible consequences (6 of 9 applications; 67%), and social control (1 of 3 applications; 33%). Across all applications, we observed resurgence during 47 of 111 schedule‐thinning transitions (42%; bottom panel), with the highest percentage when attention plus tangible consequences reinforced destructive behavior (2 of 3 transitions; 67%), followed by escape (21 of 42 transitions; 50%), tangible consequences (11 of 25 transitions; 44%), attention (10 of 27 transitions; 37%), and social control (3 of 14 transitions; 21%).

**Figure 2:**
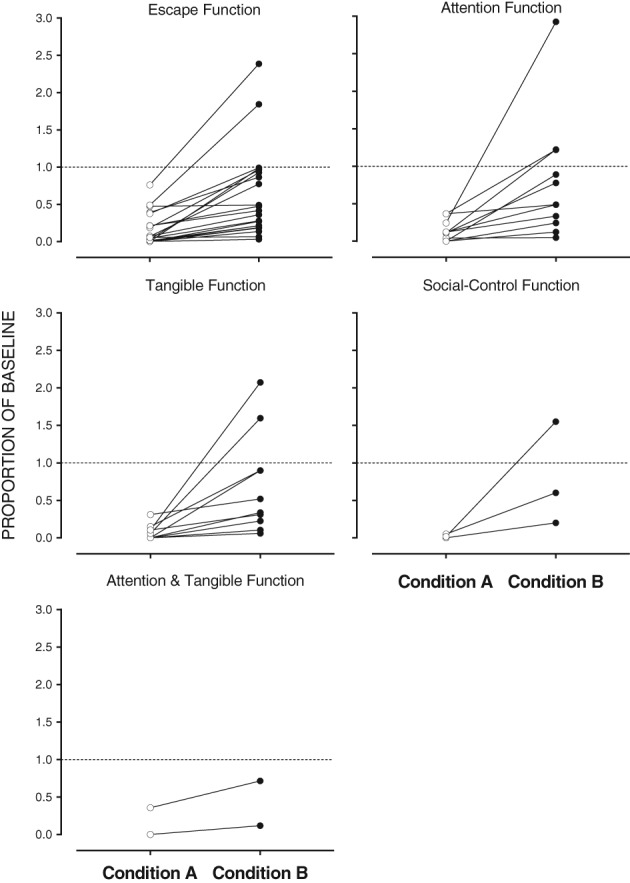
Proportion of baseline measures of resurgence during transitions across functions of destructive behavior.

Figure 2 depicts the magnitude of resurgence during transitions across functions of destructive behavior. Each line represents a transition in which we identified an instance of resurgence (transitions in which resurgence was not detected are omitted), and all data in this figure are presented as a proportion of the baseline mean. The magnitude of resurgence is depicted in two ways. First, we compared the highest rate from the last five sessions of Condition A (open circles) to the highest rate from the first three sessions of Condition B (closed circles). The average proportional response rate in Condition A across all transitions with resurgence (regardless of function) was 0.13 (an 87% reduction from baseline). By contrast, the average proportional response rate in Condition B was 0.66 (only a 44% reduction from baseline). In other words, destructive behavior increased by an average of 508% from Condition A to Condition B when comparing the highest proportional response rates in each condition. Second, we examined the number of occasions for which proportional response rates exceeded mean baseline rates (indicated by the dashed, horizontal lines in Figure 2). That is, proportional rates of 1.0 are equal to the mean baseline rate for that application; those above 1.0 represent instances of resurgence greater than the mean baseline rate for that application. Across the 19 applications in which we observed resurgence, we observed resurgence at or above a proportional rate of 1.0 in 6 (32%) of those applications. Further, across the 47 schedule‐thinning transitions in which we observed resurgence, resurgence occurred at or above a proportional rate of 1.0 in 8 (17%) of those transitions. Although we separated the resurgence data across functions, the varying number of applications within each function preclude us from making direct comparisons between functions.

## DISCUSSION

We evaluated the prevalence and characteristics of resurgence of destructive behavior during 25 applications of reinforcement schedule thinning implemented as a component of FCT. We observed resurgence in 19 of the 25 (76%) applications of reinforcement schedule thinning and in 47 of the 111 (42%) individual steps of reinforcement schedule‐thinning transitions. These results suggest that resurgence of destructive behavior is a common behavioral phenomenon when the reinforcement schedule for the appropriate alternative response (i.e., the FCR) is progressively thinned during FCT. And yet, it is important to thin the reinforcement schedule to render the treatment more practical for routine caregivers to implement.

The current results suggest that increased exposure to periods in which the FCR does not produce reinforcement during schedule thinning is a common and potentially important risk factor for treatment relapse. Nevertheless, in all cases, destructive behavior resurged temporarily and then decreased, suggesting that the resurgence of destructive behavior was a transient phenomenon during schedule thinning. Moreover, Greer, Fisher, Saini, et al. (2016) reached the target schedule (i.e., S^Δ^ periods lasting at least 4 min) in almost all applications. However, it should be noted that these researchers conducted schedule thinning in a structured treatment setting with highly trained behavior therapists. Thus, instances of resurgence may result in more sustained increases in destructive behavior when routine caregivers implement reinforcement schedule thinning, because they may be less likely to implement extinction with high integrity during such periods. That is, caregivers may be more likely than highly trained behavior therapists to deliver the functional reinforcer in response to an instance of resurgence, which would likely sustain resurgence (Bruzek, Thompson, & Peters, 2009; Mitteer, Greer, Fisher, Briggs, & Wacker, in press; St. Peter Pipkin, Vollmer, & Sloman, 2010). Future research should examine whether and to what extent caregivers show decreased procedural integrity when implementing reinforcement thinning steps that produce resurgence relative to thinning steps that do not evoke resurgence.

Lerman and Iwata (1995) found that when researchers supplemented extinction with differential reinforcement or other procedures, the prevalence of extinction bursts decreased from 36% (extinction alone) to 12% (extinction plus supplemental procedures). We found the prevalence of resurgence during schedule thinning with FCT (76%) to be much higher than the prevalence of extinction bursts reported by Lerman and Iwata. One possible reason is that reinforcement schedule thinning involves the introduction of increasingly longer periods in which extinction is implemented alone and differential reinforcement is unavailable (i.e., longer S^Δ^ periods). By contrast, when differential reinforcement interventions are first introduced, alternative reinforcement is typically available throughout. Several studies suggest that minimizing exposure to the establishing operation for destructive behavior when differential reinforcement interventions are introduced may produce more immediate reductions in destructive behavior and prevent or mitigate dangerous instances of extinction‐induced generative responding (DeRosa et al., 2015; Fisher et al., 2018). Similarly, researchers have attempted to mitigate resurgence by controlling the exposure to the establishing operation by either providing reinforcement on a fixed‐time schedule (Lieving & Lattal, 2003, Experiment 3; Marsteller & St. Peter, 2014) or by selecting the initial schedule densities during schedule thinning based on patterns of prior responding such as (a) latency to destructive behavior (e.g., Lalli, Casey, & Kates, 1997), (b) mean interresponse times for destructive behavior (e.g., Kahng, Iwata, DeLeon, & Wallace, 2000), (c) results of a progressive‐interval assessment (Fisher, Greer, Fuhrman, Saini, & Simmons, in press; Fisher et al., 2018), or (d) rate of mands (Call et al., 2017). Future research is warranted to determine whether these or similar procedures might mitigate resurgence of destructive behavior by minimizing initial exposure to the establishing operation for the reinforcer at the start of FCT schedule thinning (Saini et al., 2016; Shamlian et al., 2016).

Additionally, it may be that the effects of increased exposure to the establishing operation differ across functions of destructive behavior. Specifically, reinforcement schedule thinning for social‐positive reinforcement (e.g., multiple‐schedule FCT) typically involves increasing the duration of the S^Δ^ component by an arbitrary amount of time. Alternatively, schedule thinning for social‐negative reinforcement (e.g., chained‐schedule FCT) typically involves increasing the S^Δ^ component by requiring either toleration of longer periods with instruction or compliance with additional instructions. The difference between the schedule thinning approaches across social‐positive and social‐negative reinforcement might have contributed to our finding of higher prevalence of resurgence for escape‐maintained destructive behavior (i.e., 8 of 8 applications; 100%) as compared to destructive behavior maintained by social‐positive reinforcement (i.e., 10 of 14 applications; 71%) or social control (i.e., 1 of 3 applications; 33%). However, the present study included a limited number of applications unevenly distributed across functions. A larger sample of applications equally distributed across functions would need to be analyzed to determine the prevalence of resurgence across functions of destructive behavior.

A second possible reason for the prevalence of resurgence in this study versus the prevalence of bursting observed by Lerman and Iwata (1995) is that we defined resurgence relative to the rates of destructive behavior observed in the prior schedule‐thinning step (i.e., treatment), whereas Lerman and Iwata defined an extinction burst relative to the levels of destructive behavior observed during baseline. That is, we designed our definition of resurgence to be parallel to, but not equivalent to, Lerman and Iwata's definition of an extinction burst. Thus, extinction bursts may be relatively uncommon in part because the definition requires that responding increase above the highest levels observed during baseline, when the destructive behavior produced reinforcement. By contrast, resurgence may be relatively common in part because the definition requires only that responding increase above the highest levels observed during the prior treatment phase or schedule‐thinning transition. Future research might consider reviewing the strategies previous studies have used to operationally define, quantify, and measure instances of resurgence to determine the most sensitive method for capturing instances of resurgence (see Lattal et al., 2017, for a recent review of several different definitions of resurgence). Nevertheless, we observed increases in destructive behavior to clinically unacceptable levels on many occasions. These results suggest that clinicians should anticipate and be prepared to respond to momentary increases in destructive behavior during schedule thinning in a manner that protects the client, staff, and the environment. Therefore, clinicians should consider (a) oversight by appropriate professionals (e.g., Board Certified Behavior Analysts), and the use of (b) a safe treatment environment (e.g., padded surfaces and soft stimuli; Hanley, 2012), (c) session termination criteria (e.g., when minor tissue damage such as reddening of the skin or bleeding occurs; Betz & Fisher, 2011), (d) staff trained to perform minor first aid and recognize when further medical assistance is needed, and (e) protective equipment (Fisher, Rodriguez, Luczynski, & Kelley, 2013) to manage momentary increases in destructive behavior during schedule thinning.

The findings reported by Lerman and colleagues (Lerman & Iwata, 1995; Lerman et al., 1999) show that supplemental procedures like differential reinforcement can, in the majority of cases, prevent two important side effects of extinction, bursting and extinction‐induced aggression. However, the current findings indicate that differential reinforcement alone may be much less effective at preventing resurgence of destructive behavior during reinforcement schedule thinning. When overall rates of alternative reinforcement are reduced and the alternative response contacts extinction during schedule thinning, these conditions set the occasion for resurgence of destructive behavior. This finding suggests that we need to explore other strategies for conducting reinforcement schedule thinning that may reduce the likelihood of resurgence. For instance, Saini et al. (2016) suggested that providing access to alternative activities during the S^Δ^ component (e.g., Hagopian, Contrucci Kuhn, Long, & Rush, 2005) or implementing punishment across both multiple‐schedule components (e.g., Hagopian, Bruzek, Bowman, & Jennett, 2007; Kestner, Redner, Watkins, & Poling, 2015) might be strategies researchers investigate in the future to reduce the prevalence of resurgence during schedule thinning.

Another approach to preventing or mitigating resurgence of destructive behavior, based on behavioral momentum theory (BMT), involves one or more of the following modifications to decrease the momentum of destructive behavior: (a) decreasing the rates of reinforcement in baseline, (b) decreasing the rates of alternative reinforcement during FCT, (c) lengthening the duration of FCT prior to exposing the FCR to periods of extinction, (d) rendering transitions from baseline to treatment reinforcement contingencies highly salient and transitions from treatment reinforcement contingencies to extinction highly indiscriminable, and (e) altering the stimulus context (for discussions, see Greer, Fisher, Romani, & Saini, 2016; Nevin & Shahan, 2011; Podlesnik & DeLeon, 2015; Podlesnik, Kelley, Jimenez‐Gomez, & Bouton, 2017; Shahan & Sweeney, 2011). Future research should continue to examine whether modifications informed by BMT (e.g., Fisher et al., in press; Fisher et al., under review; Fuhrman et al., 2016; Saini & Fisher, 2016; Sweeney & Shahan, 2013) or other theories of resurgence (e.g., resurgence as choice; Shahan & Craig, 2017) should be considered for informing future translational and applied investigations aimed at developing procedures for mitigating resurgence.

In summary, the current findings clearly establish that resurgence of destructive behavior is a common behavioral phenomenon during reinforcement schedule thinning and suggest that the prevalence of resurgence varies according to the function of destructive behavior. Overall, these findings suggest several possible avenues of future investigation that may help to elucidate the variable(s) that promote and diminish resurgence of destructive behavior.
